# Synergistic and independent action of endogenous microRNAs 122a and 199a for post-transcriptional liver detargeting of gene vectors

**DOI:** 10.1038/s41598-018-33801-4

**Published:** 2018-10-19

**Authors:** Bijay Dhungel, Charmaine A. Ramlogan-Steel, Jason C. Steel

**Affiliations:** 1 Gallipoli Medical Research Institute, Greenslopes Private Hospital, 102 Newdegate Street, Brisbane, 4120 QLD Australia; 2 Faculty of Medicine, The University of Queensland, 288 Herston Road, Herston, Brisbane, 4006 QLD Australia; 3 University of Queensland Diamantina Institute, Translational Research Institute, 37 Kent Street, Woolloongabba, 4102 QLD Australia; 4 CQUniversity, Bruce Highway, North Rockhampton, 4702 QLD Australia

## Abstract

In hepatocellular carcinoma (HCC), which usually develops in a cirrhotic liver, treatments preserving normal liver function and viability are vitally important. Here, we utilise the differential expression of miRNAs 122a and 199a between normal hepatocytes and HCC to generate vectors harbouring their binding sites for hepatocyte detargeting. Using a reporter gene, we observed a synergistic detargeting of cells expressing both miRNAs as well as cells expressing either of the miRNAs; while expression was retained in HCC cells negative for both miRNA122a and miRNA199a. Mimics and inhibitors for individual miRNAs were used to confirm these results. Furthermore, suicide gene therapy with cytosine deaminase (CD)/5-fluorocytosine system resulted in limited killing of cells expressing either of the two miRNAs. Finally, we report feasibility of using adeno associated virus (AAV) based vectors for delivery of this dual regulated gene delivery system. These results present a novel dual targeted system whereby miRNA122a and miRNA199a act either synergistically or independently in regulating transgene expression with vectors harbouring binding sites of both miRNAs and have implications in detargeting vectors from multiple cell types in the liver.

## Introduction

Hepatocellular carcinoma (HCC), the main form of primary liver cancer, is the third highest cause of cancer related mortality globally^1^. The majority of HCC develop in a background of chronic liver disease and cirrhosis where systemic chemotherapy and radiation therapy are of limited benefit due to a high incidence of side effects, poor efficacy, and dose-limiting hepatotoxicity in the setting of cirrhosis^2,3^. While liver transplant and resection can be curative in an early stage, most patients are diagnosed at a late stage where the therapeutic options are poor^4^. Given the poor liver condition of most HCC patients, there is a need to develop targeted liver sparing therapies. Targeted gene therapy may provide a platform for treating HCC. Gene therapy approaches for treating diseases have become a reality with recent approval of adeno associated virus (AAV) based drugs by both the European Union and the US Food and Drug Administration^3,5,6^. Cancer targeting can be achieved either by transductional targeting by modifying delivery vectors^7^ or using gene regulatory elements like tumor specific promoters and miRNAs that are dysregulated in cancer^5,8,9^.

microRNAs (miRNAs) are small RNA molecules which regulate the expression of a gene at the post-transcriptional level. It does this by interacting through complementary base-pairing with a specific binding site/target site (TS) in the corresponding messenger RNA thereby altering its stability and translation. A number of studies have reported efficient detargeting of a cell/tissue by inclusion of TSs of endogenous microRNAs at the 3′-UTR of a transgene^8,10–12^. Given that several miRNAs are downregulated in HCC while being expressed at high levels in the liver (off-target cells)^13–15^, inclusion of TSs of these miRNAs could provide an effective means of regulating the expression of therapeutic cargo in HCC cells while sparing normal liver cells.

In this proof-of-concept study, we demonstrate that multiple liver miRNA TSs can be incorporated into a gene delivery vector to detarget cells of the liver thereby allowing targeted gene therapy. First, we identified miRNAs expressed in primary hepatocytes but not in HCC cell lines. We observed a downregulation of miRNA122a and miRNA199a in HCC cells except for HuH7 which expresses high levels of miRNA122a and Hepa1–6 which has endogenous miRNA199a at levels comparable to primary human hepatocytes. Using vectors in which we included the TSs of both miRNA122a and miRNA199a we show significant inhibition of reporter expression in both HuH7 and Hepa1-6 and little reduction in HCC cell lines which do not express either miRNA. We also validate the role of these miRNAs in the targeting of HCC using miRNA mimics and inhibitors. Furthermore, we explore the feasibility of using these vectors to limit the effects of the suicide gene cytosine deaminase (CD) to HCC cell lines.

Our results suggest that post-transcriptional targeting of HCC can be achieved by a vector containing multiple miRNA TSs whereby the endogenous miRNAs act either independently or in synergy to limit transgene expression. These results (summarized in Supplementary Fig. 1) have implications for multiple cell type detargeting as miRNA122a is hepatocyte specific whereas miRNA199a is expressed by other cell types in the liver including hepatic stellate cells and liver sinusoidal epithelial cells.

## Results

### miRNA122a and miRNA199a are downregulated in HCC

**Figure 1:**
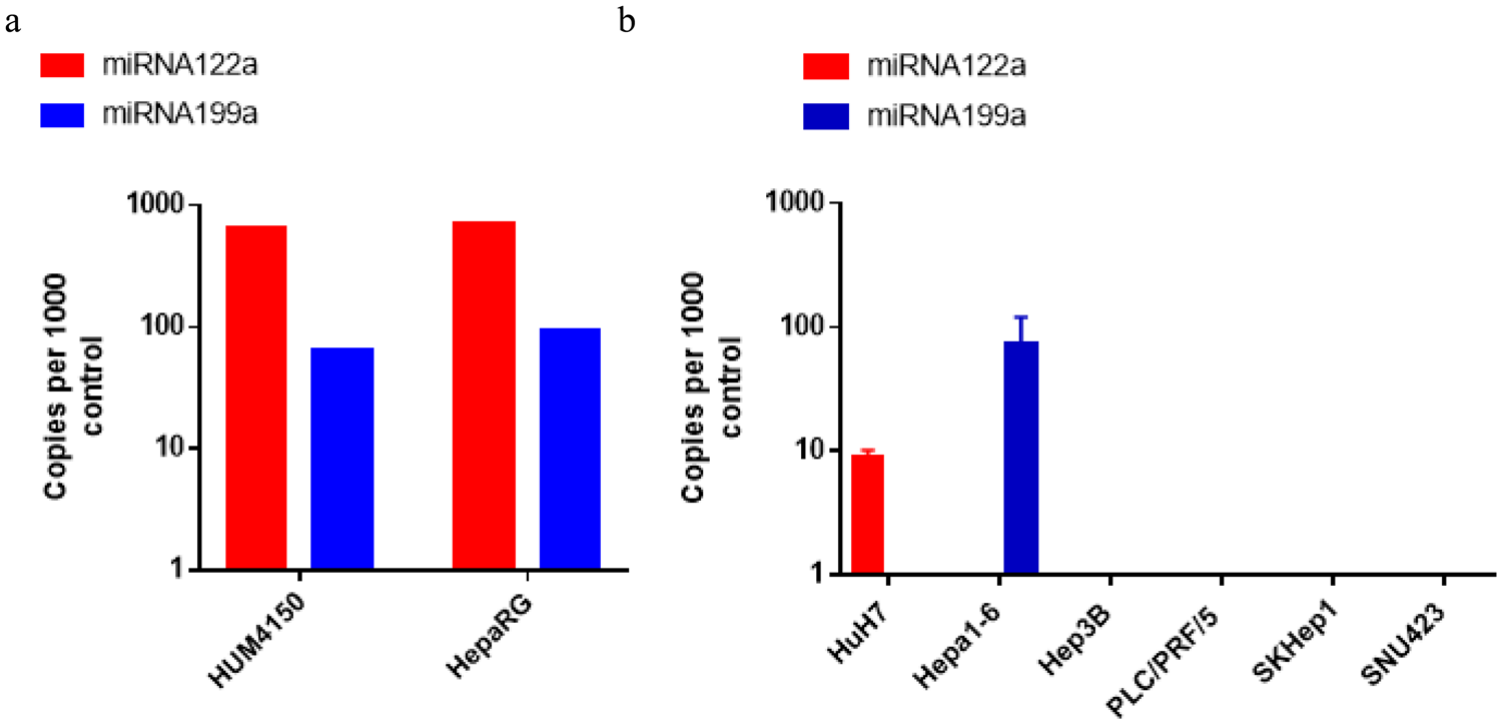
Expression levels of miRNA122a and miRNA199a: qRT-PCR was performed on total RNA extracted from primary hepatocytes (**a**) and HCC cell lines (**b**) to calculate the expression levels of miRNA122a and miRNA199a. Copies of each miRNA was calculated as copies per 1000 RNU6 control. (n > 3).

To study the expression profile of miRNA122a and 199a in hepatocytes and HCC, we performed real time quantitative PCR on cDNA obtained from primary hepatocytes (Fig. 1a) and HCC (Fig. 1b) cell lines. After normalizing against the RNU6 control primer, we detected high endogenous levels of miRNA122a in both primary hepatocytes HUM4150 and HepaRG (641 and 700 copies per 1000 RNU6). The expression of miRNA199a was detected to be 9.8 and 7.5-fold lower than miRNA122a in HUM4150 and HepaRG respectively. Both the miRNAs were undetected in HCC cell lines Hep3B, PLC/PRF/5, SKHep1, and SNU423. miRNA122a expression was detected in HuH7 cells (71 and 77 folds lower than HUM4150 and HepaRG respectively). Similarly, 74 copies of miRNA199a per 1000 copies of RNU6 was detected in Hepa1-6 cells which was similar to expression levels detected in HUM4150 (65 copies per 1000 control) and HepaRG (93 copies per 1000 control). These results suggested that both miRNA122a and miRNA199a are downregulated in HCC cell lines used in this study except for HuH7 (positive for miRNA122a) and Hepa1-6 (positive for miRNA199a).

### Endogenous miRNA122a and miRNA199a can be exploited independently for detargeting cells by incorporating binding sites of both at the 3′-UTR of a transgene

**Figure 2:**
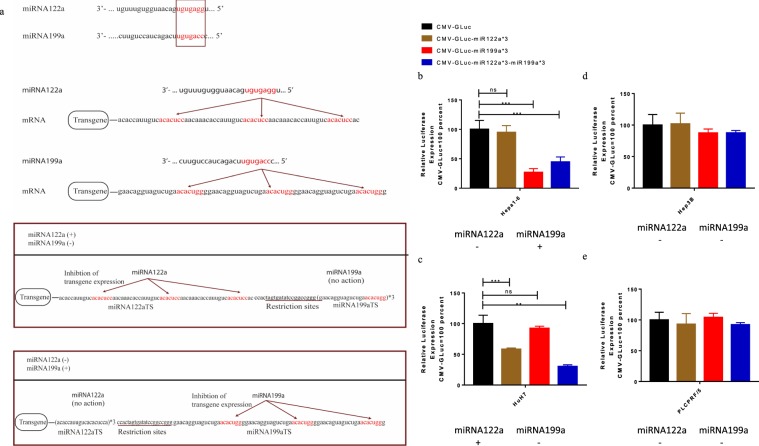
Post-transcriptional regulation of transgene expression in HCC cells by endogenous miRNA122a and miRNA199a. (**a**) *Mechanisms of action of dual regulated expression vector based on miRNA122a and miRNA199a:* Vectors with binding sites (TSs) of both miRNA122a and miRNA199a can detarget cells which have high endogenous levels of either miRNA122a, miRNA199a, or both. The seed sequences are shown in red. Plasmids with TSs of either miRNA122a, miRNA199a, or both miRNA122a and miRNA199a were used to transfect miRNA199a positive Hepa1-6 (**b**), miRNA122a positive HuH7 (**c**), and double negative HCC cell lines Hep3B (**d**) and PLC/PRF/5 (**e**). The amount of secreted Gluc for each group was normalized with CMV-Gluc control for each cell line. The observed differences between CMV-Gluc and each group was tested for statistical significance by performing two tailed t-test using Graph Pad Prism 7.0. (n > 3, ***p < 0.005, **p < 0.01).

To investigate whether an expression vector containing TSs of both miRNA122a and miRNA199a could be effective in detargeting different cell types with high endogenous levels of either of the two miRNAs, we constructed different expression plasmids harbouring Gaussia Luciferase (Gluc) with TSs of miRNA122a (CMV-Gluc-miR122a*3), miRNA199a (CMV-Gluc-miR199a*3), and of both miRNA122a and miRNA199a (CMV-Gluc-miR122a*3-miR199a*3). miRNA122a positive HuH7 cells, miRNA199a positive Hepa1-6 cells, and HCC cell lines negative for both the miRNAs were transfected with these plasmids and the observed Gluc expression for each group was normalized with that observed after transfection with CMV-Gluc control plasmid. Hepa1-6 transfection with CMV-Gluc-miR199a*3 and CMV-Gluc-miR122a*3-miR199a*3 resulted in a 3.7 and 2.2-fold inhibition of Gluc expression respectively (p < 0.005), whereas expression was retained when Hepa1-6 cells were transfected with CMV-Gluc-miR122a*3 compared to the CMV-Gluc control (Fig. 2b). Similarly, a significant reduction in Gluc expression was observed in HuH7 cells transfected with CMV-Gluc-miR122a*3 (p < 0.005) and CMV-Gluc-miR122a*3-miR199a*3 (p < 0.01) but no significant changes were observed with CMV-Gluc-miR199a*3 compared to the CMV-Gluc control (Fig. 2c). No significant inhibition of the reporter was observed in HCC cell lines Hep3B (Fig. 2d) and PLC/PRF/5 (Fig. 2e) and non-HCC cell lines (Supplementary Fig. 2) transfected with either of the plasmids. Taken together, these results indicate that TSs of miRNA122a and miRNA199a present in the same expression vector can independently be used to inhibit the expression of transgene on different cell types with high endogenous levels of either of the two miRNAs while retaining expression in HCC cells in which these miRNAs are low or absent.

### Inclusion of multiple miRNA target sites can function independently and synergistically to detarget cells

To confirm the role of miRNA expression on the detargeting of our vectors, we utilized miRNA122a and miRNA199a inhibitors and mimics to alter miRNA expression in Hepa1-6 (miRNA199a expressing) and HuH7 (miRNA 122a expressing) cell lines.

**Figure 3:**
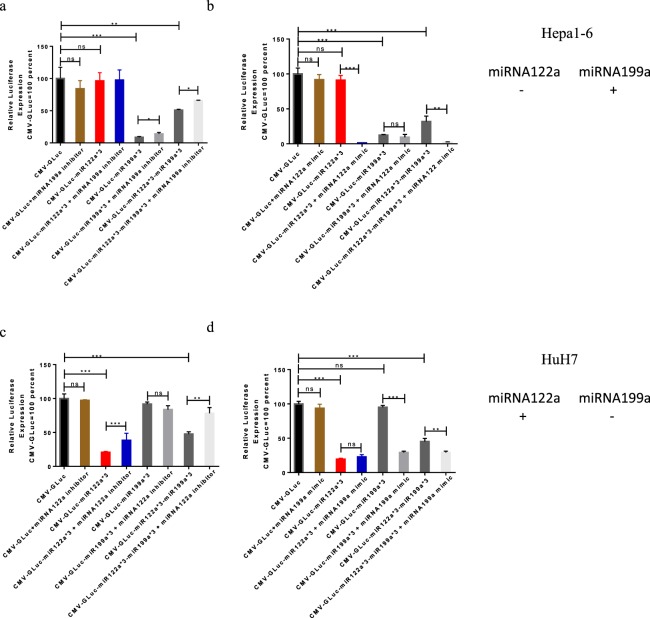
Alteration of endogenous miRNA122a and miRNA199a in Hepa1-6 and HuH7. (**a**) *Inhibition of miRNA199a in Hepa1-6:* Hepa1-6 were co-transfected with miRNA199a inhibitor and either CMV-Gluc, CMV-Gluc-miR122a*3, CMV-Gluc-miR199a*3, or CMV-Gluc-miR122a*3-miR199a*3 following which the amount of secreted Gluc was calculated; secreted Gluc from each group was normalized against that observed after transfection with CMV-Gluc (**b**) *Overexpression of miRNA122a in Hepa1-6:* Co-transfection of miRNA122a mimic and either CMV-Gluc, CMV-Gluc-miR122a*3, CMV-Gluc-miR199a*3, or CMV-Gluc-miR122a*3-miR199a*3 was performed in Hepa1-6 and the amount of secreted Gluc was expressed as a percentage of Gluc expressed after transfection with CMV-Gluc (**c**) *Inhibition of miRNA122a in HuH7:* HuH7 cells were co-transfected with miRNA122a inhibitor and either CMV-Gluc, CMV-Gluc-miR122a*3, CMV-Gluc-miR199a*3, or CMV-Gluc-miR122a*3-miR199a*3 and the amount of secreted Gluc was calculated; secreted Gluc from each group was normalized against that with CMV-Gluc (**b**) *Overexpression of miRNA199a in HuH7:* Co-transfection of miRNA199a mimic and either CMV-Gluc, CMV-Gluc-miR122a*3, CMV-Gluc-miR199a*3, or CMV-Gluc-miR122a*3-miR199a*3 was performed in HuH7 and the amount of secreted Gluc was expressed as a percentage of Gluc expressed after transfection with CMV-Gluc. (n > 3, *p < 0.05, **p < 0.01, ***p < 0.005).

When using miRNA199a inhibitor to reduce the endogenous levels of miRNA199a in Hepa1-6 cells followed by transfection with either CMV-Gluc-miR199a*3 or CMV-Gluc-miR122a*3-199a*3 we noted significantly increased expression of Gluc compared to uninhibited Hepa1-6 cells transfected with either CMV-Gluc-miR199a*3 or CMV-Gluc-miR122a*3-miR199a*3 alone (p < 0.05) (Fig. 3a). When the levels of miRNA122a in Hepa1-6 were increased using a miRNA122a mimic, we noted significant decreases in Gluc expression following transfection with either CMV-Gluc-miR122a*3 or CMV-Gluc-miR122a*3-miR199a*3 as compared to unmodified Hepa1-6 cells transfected with CMV-Gluc-miR122a*3 or CMV-Gluc-miR122a*3-miR199a*3 (p < 0.01) (Fig. 3b).

Similarly, the inhibition of miRNA122a in HuH7 resulted in an increase of Gluc expression by 1.9 and 1.6-fold after transfection with CMV-Gluc-miR122a*3 (p < 0.005) or CMV-Gluc-miR122a*3-miR199a*3 (p < 0.01) respectively, whereas no significant changes were observed in reporter expression after transfection with CMV-Gluc-miR199a*3 (Fig. 3c). HuH7 co-transfection with miRNA199a mimic and either CMV-Gluc-miR199a*3 or CMV-Gluc-miR122a*3-miR199a*3 resulted in a significant reduction of Gluc expression (p < 0.005 and p < 0.01 respectively) whereas no significant change in Gluc expression was observed after co-transfection of HuH7 with miRNA199a mimic and CMV-Gluc-miR122a*3 (Fig. 3d). These results indicate that inhibition of Gluc expression observed after transfection of CMV-Gluc-miR122a*3-miR199a*3 in both HuH7 and Hepa1-6 is the result of independent action of miRNA122a (in HuH7) and miRNA199a (in Hepa1-6). Moreover, the use of miRNA mimics suggests that detargeting with our system can also be synergistic when both miRNAs are expressed at adequate levels in the same cell.

### Post-transcriptionally regulated suicide gene therapy system based on gene regulation mediated by both miRNA122a and miRNA199a

**Figure 4:**
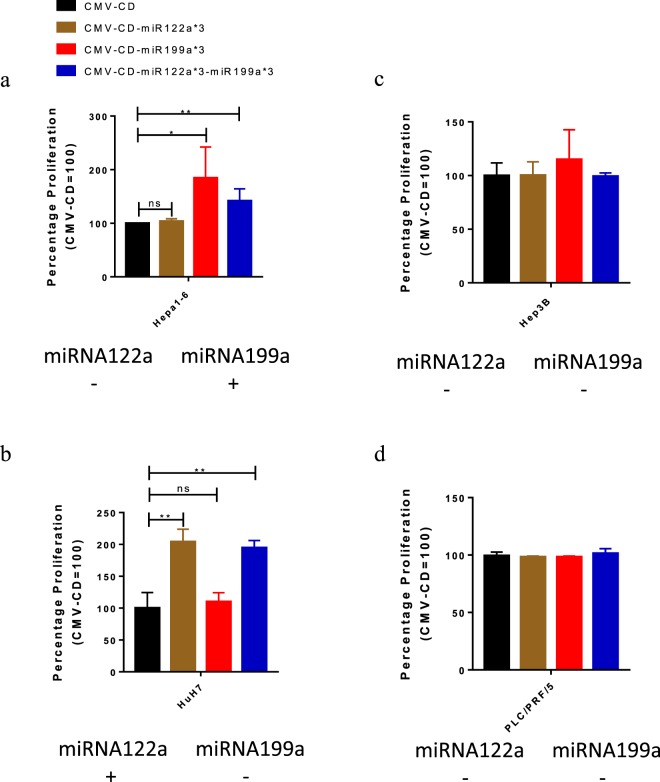
Post transcriptionally targeted suicide gene therapy for HCC. To study the feasibility of performing suicide gene therapy with the dual-regulated system, HCC cell lines Hepa1-6 (a), HuH7 (b), Hep3B (c), and PLC/PRF/5 (d) were transfected with either CMV-CD, CMV-CD-miR122a, CMV-CD-miR199a*3, or CMV-CD-miR122a*3-miR199a*3. After incubation with the prodrug 5-fluorocytosine (5-FC), percentage proliferation was calculated with MTS assay. Proliferation percentage of cells transfected with CMV-CD-miR122a, CMV-CD-miR199a*3, or CMV-CD-miR122a*3-miR199a*3 and incubation with 5-FC was normalized with percentage proliferation observed after suicide gene therapy with CMV-CD for each cell line. (n > 3, * < 0.05, **p < 0.01).

After establishing the independent action of miRNA122a and miRNA199a in the regulation of our dual targeted vector, we explored the possibility of delivering the suicide gene CD with this system (Fig. 4). miRNA122a positive HuH7, miRNA199a positive Hepa1-6 and double negative HCC cell lines Hep3B and PLC/PRF/5 were transfected with CMV-CD, CMV-CD-miR122a*3, CMV-CD-miR199a*3, and CMV-CD-miR122a*3-miR199a*3. After incubation of the transfected cells with prodrug 5-FC, we observed a significantly higher proliferation of Hepa1-6 transfected with CMV-CD-miR199a*3 (p < 0.05) as well as CMV-CD-miR122a*3-miR199a*3 (p < 0.01) when compared to CMV-CD (Fig. 4a). The greater proliferation in these cells correlated with decreased killing by CD. In contrast, there was no differences in proliferation of Hepa1-6 cells treated with CMV-CD or CMV-CD- miRNA122a*3, indicating no inhibition of killing in the cells treated with CMV-CD- miRNA122a*3 compared to CMV-CD. Similarly, HuH7 cells transfected with either CMV-CD-miR122a*3 (p < 0.05) or CMV-CD-miR122a*3-miR199a*3 (p < 0.05) and incubated with 5-FC showed significantly higher proliferation when compared to cells transfected with CMV-CD (Fig. 4b). Whereas, CMV-CD-miRNA199 exhibited no significant changes in proliferation when compared to the unregulated CMV-CD control.

When comparing the effects of CMV-CD with CMV-CD-miR122a*3, CMV-CD-miR199a*3, or CMV-CD-miR122a*3-miR199a*3 in HCC cell lines that had low or little miRNA199a or miRNA122a, i.e. Hep3B and PLC/PRF/5 we saw no inhibition in CD induced killing (Fig. 4c,d). These results suggest that like regulated reporter expression, our system is able to delivery post transcriptionally regulated suicide gene therapy for HCC based on actions of both miRNA122a and miRNA199a.

### Adeno associated virus mediated delivery of transgene regulated synergistically and independently by miRNA122a and miRNA199a for HCC-targeted gene delivery

**Figure 5:**
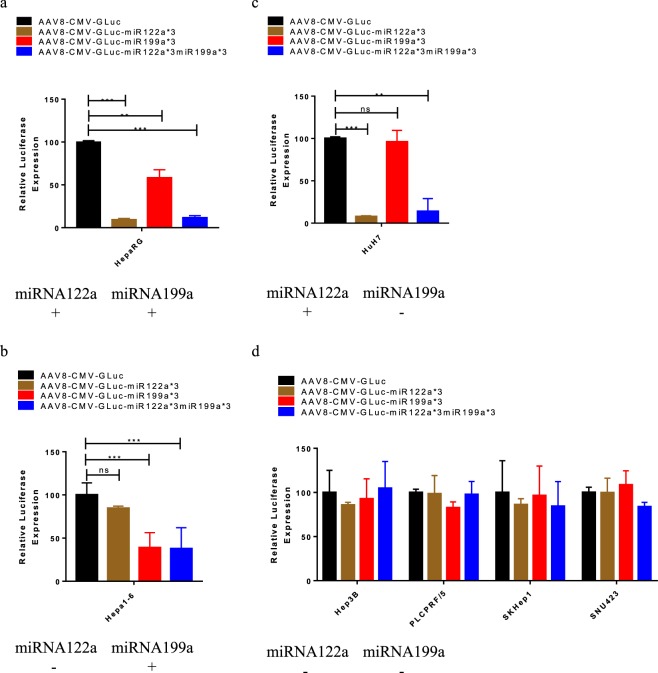
Adeno associated virus mediated delivery of dual post-transcriptionally regulated gene delivery system. AAV8-CMV-Gluc-miR122a*3, AAV8-CMV-Gluc-miR199a*3, and AAV8-CMV-Gluc-miR122a*3-miR199a*3 were used to transduce primary hepatocyte HepaRG (**a**), Hepa1-6 (**b**), HuH7 (**c**), and HCC cell lines Hep3B, PLC/PRF/5, SKHep1, and SNU423 (**d**). The amount of secreted Gluc was measured for each group and expressed as a percentage of Gluc expression measured after transduction of the corresponding cell with AAV8-CMV-Gluc. (n > 3, **< 0.01, ***< 0.005).

To study the feasibility of vectorizing our dual HCC-targeted constructs, we developed AAV8 based vectors with reporter Gluc containing TSs of miRNA122a (AAV8-CMV-Gluc-miR122a*3), miRNA199a (AAV8-CMV-Gluc-miR199a*3), and both miRNA122a and miRNA199a (AAV8-CMV-Gluc-miR122a*3-miR199a*3). Here, we expanded our panel of cells to include the primary hepatocyte line HepaRG (positive for both miRNAs), Hepa1-6 (positive for miRNA199a), HuH7 (positive for miRNA122a) and the HCC cell lines Hep3B, PLC/PRF/5, SKHep1 and SNU423 (negative for both miRNAs). The cell lines were transduced with these AAV vectors and the amount of secreted Gluc was normalized against that secreted after transduction with AAV8-CMV-Gluc. In the normal hepatocyte line, HepaRG, all 3 miRNA regulated vectors exhibited a significant reduction in expression when compared to AAV8-CMV-Gluc with AAV8-CMV-Gluc-miR122a*3 (p < 0.005), AAV8-CMV-Gluc-miR199a*3 (p < 0.05), or AAV8-CMV-Gluc-miR122a*3-miR199a*3 (p < 0.005) (Fig. 5a). Hepa1-6 transduced with AAV8-CMV-Gluc-miR122a*3 lead to no significant reduction in Gluc expression, whereas significantly inhibited Gluc expression was observed after transduction with AAV8-CMV-Gluc-miR199a*3 (p < 0.005) and AAV8-CMV-Gluc-miR122a*3-miR199a*3 (p < 0.005) (Fig. 5b). In the case of HuH7 cells, inhibition of reporter expression was observed after transduction with AAV8-CMV-Gluc-miR122a*3 (p < 0.005) and CMV-Gluc-miR122a*3-miR199a*3 (p < 0.005) but not with AAV8-CMV-Gluc-miR199a*3 (Fig. 5c). In the miRNA199a and 122a negative HCC cell lines (Hep3B, PLCPRF/5, SKHep1 and SNU-423), there was no reduction in transgene expression for any of the miRNA regulated vectors when compared to the unregulated AAV8-CMV-Gluc control.

## Discussion

Targeted cancer gene therapy requires therapeutic genes to be expressed in cancer cells while minimal expression is expected in normal cells/tissues. miRNAs are powerful regulators of gene expression which act by binding to their target sequence in the corresponding mRNA and either inhibiting translation and/or degrading the mRNA^16^. Exploiting miRNAs which are expressed at high levels in normal cells while being downregulated in HCC by inclusion of their TSs at the 3′-UTR of a transgene is, thus, an effective method for restricting off-target effects. In this study, we used miRNA122a and miRNA199a, both expressed at high levels in liver and downregulated in number of HCC, for targeted gene delivery in HCC cells.

miRNA122a is the most abundant miRNA in liver^17,18^ whereas miRNA199a is amongst the most highly expressed^19^ and both have been reported to be downregulated in HCC^17,19–21^. In fact, both these miRNAs have been studied as potential therapeutic targets as well as biomarkers for HCC^14,22,23^. In line with these reports, we also observed a significant reduction in miRNA199a and miRNA122a in most of our HCC cell lines Hep3B, PLC/PRF/5, SKHep1, and SNU423. From our panel of HCC lines, HuH7 (positive for miRNA122a) and Hepa1-6 (positive for miRNA199a) were the outliers. The expression of miRNA122a and miRNA199 by HuH7 cells^24–26^ and Hepa1-6 cells^27^, respectively, has been previously reported. The identification of cells with differing miRNA 199a and miRNA122a is important when establishing a liver model, particularly given the heterogeneity of the liver environment and the need to detarget multiple cell types in the liver and allowed us to model our dual targeted vectors in cells expressing either miRNA singularly. The existence of HCC lines with variable expression of miRNA species is not unexpected due to the well-known molecular heterogeneity of HCC in relation to its differing etiology, including different liver carcinogens, viruses, and genetic background. Hepatocytes have been shown here by us and previously by others to express both miRNA122a and miRNA199a^14,17,28^, whereas, miRNA199a is expressed by other major liver cells including hepatic stellate cells^29^ and liver sinusoid endothelial cells^30^. By incorporating the TSs of both miRNA122a and miRNA199a into our vectors we attempted to show that cells expressing either miRNA could be detargeted. Indeed, our results suggested that in our dual-targeted gene regulation system, these two miRNAs TSs could act synergistically in hepatocytes which express both the miRNAs and independently in cells which express either miRNA122a (HuH7) or miRNA199a (Hepa1-6). Similarly, given that previous studies have incorporated miRNA122a TSs in vectors to detarget liver and target different tissues including adipose tissue^31^, dendritic cells^32^, central nervous system^33^, and fibrosarcoma xenografts^34^, it may be feasible to target other cancer types with a downregulation of miRNA199a, including breast cancer^35^, renal cell cancer^36^, osteosarcoma^37^, thyroid cancer^38^ and bladder cancer^39^.

In this study, we compared the seed sequence between miRNA122a and miRNA199a and noted a homology of 5 bases out of 7 for this region. To examine whether this homology would result in cross targeting of the miRNA, we co-transfected Hepa1-6 with miRNA122a mimic and CMV-Gluc-miR199a*3 and showed that the combination did not alter the reporter expression when compared to transfection with CMV-Gluc-miR199a alone, suggesting that despite homology of positions 4–8 the binding of miRNA122a and 199a to their corresponding TSs is mutually exclusive.

The feasibility of inhibiting suicide gene therapy with this system adds to the possibility of sparing non-HCC liver cells and preventing off-target effects. Furthermore, our results suggest that it would be plausible to combine this dual detargeted post-transcriptionally gene regulated system with other targeting strategies including transductional and transcriptional targeting using the AAV vector system. Furthermore, it could be possible to incorporate multiple miRNAs into the vectors to detarget multiple tissues given the small size of their binding sites, however, consideration should be given to the accessibility of binding sites which could be blocked by the structural complexity often observed in longer mRNAs^40^.

In conclusion, we report a post-transcriptionally regulated transgene delivery system for HCC which is regulated by two highly abundant miRNAs in the normal liver, miRNA122a and miRNA199a, but downregulated in HCC, which can act either synergistically or independently depending on the level of each miRNA in individual cell types.

## Methods

### Cell Culture

Cryopreserved primary human hepatocytes (HUM4150) and HepaRG (NSHPRG) cells were obtained from Lonza, Australia and maintained according to the manufacturer’s protocol. HCC cell lines HuH7 was provided by Dr. Kim Bridle and HCC cell lines Hepa1-6, Hep3B, PLC/PRF/5, SKHep1, and SNU423 were obtained from American Type Culture Collection (ATCC) and cultured in DMEM media (Thermo Fisher Scientific, Scoresby, Australia) supplemented with 10% fetal bovine serum (Gibco, Australia) and 1% penicillin/streptomycin (P/S) (Gibco, Australia). Cell line identification service was provided by the Australian Genome Research Facility (AGRF). Non-HCC cell lines HEK293, TOV-21G, LNCaP, and COS7 were obtained from ATCC and maintained in media mentioned above.

### Quantification of miRNA levels

Endogenous levels of miRNAs were quantified by real time quantitative PCR (qRT-PCR). Total RNA was extracted from cells using trizol and cDNA was synthesized using the MystiCq microRNA cDNA Synthesis Mix (Sigma Aldrich, St. Louis, MO, USA). Next, Bioline Lo-Rox Sybr (Bioline, Australia) was used to perform qRT-PCR in the ViiA7 RT-PCR machine (Thermo Fisher Scientific) under the following conditions: 95 °C: 10 m followed by 40 cycles of 95 °C: 5 s, 60 °C:10 s and 70 °C: 10 s. Forward primer used for miRNA122a was 5′-TGGAGTGTGACAATGGTGTTTGT-3′ whereas that for miRNA199a was 5′-CCCAGTGTTCAGACTACCTG-3′. Both the miRNAs were expressed as number of copies per 1000 copies of the control microRNA RNU6-1 (MIRCP00001) using the formula (2^ (Ct (RNU6-1)-Ct (miRNA122a/199a)) *1000. MystiCq Universal PCR (MIRUP, Sigma) was used as a reverse primer for the qRT-PCR reactions.

### Plasmid construction

The gene of Gluc was artificially synthesized by Thermo Fisher Scientific to harbour three miRNA122a and miRNA199a TSs at the 3′-UTR (either alone or in combination) (Fig. 2c). These constructs were cloned into the pscAAV-GFP plasmid (Addgene #32396) with enzymes EcoRI and StuI to obtain CMV-Gluc, CMV-Gluc-miR122a*3, CMV-Gluc-miR199a*3, and CMV-Gluc-miR122a*3-miR199a*3. Similarly, suicide gene cytosine deaminase (CD) was artificially synthesized (Thermo Fisher Scientific) and cloned into each of the constructs to replace the Gluc reporter giving CMV-CD, CMV-CD-miR122a*3, CMV-CD-miR199a*3, and CMV-CD-miR122a*3-miR199a*3.

### Transfections and luciferase reporter assay

For Gluc reporter assays, transfections were performed with Lipofectamine 3000 (Thermo Fisher Scientific) in a 24 well plate according to manufacturer’s recommendations. Cells were transfected with 500 ng of either CMV-Gluc, CMV-Gluc-miR122a*3, CMV-Gluc-miR199a*3 or CMV-Gluc-miR122a*3-miR199a*3 and the amount of secreted Gluc was quantified after 48–72 hours using the Gaussia glow assay kit (Thermo Fisher Scientific). The chemiluminescence was measured in the Infinite 200 Pro NanoQuant (Tecan Trading AG, Switzerland). Gluc expression after transfection of cells with CMV-Gluc-miR122a*3, CMV-Gluc-miR199a*3, or CMV-Gluc-miR122a*3-miR199a*3 was normalized with Gluc expression after transfection with CMV-Gluc and reported as its percentage.

### miRNA inhibition and overexpression

Individual miRNAs were inhibited and overexpressed with miRNA inhibitors and mimics (Life Technologies, Mulgrave, Australia). HuH7 cells were co-transfected with miRNA inhibitor (5pmol) (MH11012, Life Technologies, Australia) and either CMV-Gluc, CMV-Gluc-miR122a*3, CMV-Gluc-miR199a*3, or CMV-Gluc-miR122a*3-miR199a*3. To overexpress miRNA199a in HuH7 cells, co-transfection was performed with miRNA199a mimic (MC10893, Life Technologies, Australia) and either CMV-Gluc, CMV-Gluc-miR122a*3, CMV-Gluc-miR199a*3, or CMV-Gluc-miR122a*3-miR199a*3. Similarly, Hepa1-6 cells were co-transfected with miRNA199a inhibitor (MH10893, Life Technologies, Australia) and either CMV-Gluc, CMV-Gluc-miR122a*3, CMV-Gluc-miR199a*3, or CMV-Gluc-miR122a*3-miR199a*3. Overexpression of miRNA122a in Hepa1-6 cells was performed by co-transfection of miRNA122a mimic (MC11012, Life Technologies, Australia) and either CMV-Gluc, CMV-Gluc-miR122a*3, CMV-Gluc-miR199a*3, or CMV-Gluc-miR122a*3-miR199a*3. Secreted Gluc for each group was quantified 48–72 hours after transfection and normalized with the corresponding value of Gluc secreted after transfection with CMV-Gluc.

### Cell proliferation

To study cell proliferation after suicide gene therapy with CD/5-FC system, 10000 cells were transfected with 100 ng of either CMV-CD, CMV-CD-122a*3, CMV-CD-199a*3, or CMV-CD-miR122a*3-miR199a*3 in a 96-well plate and fresh media containing 10 µm 5-FC was added. After 48 hours of incubation, CellTiter96 Aqueous One Solution Cell Proliferation Assay kit (Promega Corporation, Madison, WI USA) was used to quantify cell proliferation as per manufacturer’s protocol. Absorbance at 540 nm was measured with the Infinite 200 Pro NanoQuant. Percentage proliferation after suicide gene therapy with CMV-CD-miR122a*3, CMV-CD-miR199a*3, and CMV-CD-miR122a*3-miR199a*3 was expressed as percentage proliferation after suicide gene therapy with CMV-CD.

### Adeno associated virus production and transduction

AAVs were produced by triple transfection of AAV293 cells with AAV8-RC plasmid, pHelper (Agilent Technologies, Mulgrave, Australia) and either pscCMV-Gluc, psc-CMV-Gluc-miR122a*3, pscCMV-Gluc-miR199a*3, or pscCMV-Gluc-miR122a*3-miR199a*3 in a 1:2:1 ratio. Transfections were performed in 15 cm plates and cells were collected after 48–72 hours, washed with PBS and subjected to 3 freeze thaw cycles in ethanol/dry ice and incubation at 37 °C. After centrifugation, the supernatant was collected and treated with benzonase (Sigma Aldrich, Australia) for one hour at 37 °C. The benzonase treated supernatant was then centrifuged at 3500 rpm for 25 minutes, and subsequently passed through a 0.45-micron filter. The vector was then concentrated using a 100 kDa Amicon Ultrafilter (Sigma Aldrich, Australia) and buffer exchange was performed thrice with PBS after which the final AAV prep was collected at a volume of 200–500 µl. Titration of the vector prep was then performed with qRT-PCR and expressed as number of vector genomes (vgs). A standard curve was generated from a plasmid of known concentration harbouring Gluc and absolute quantification was performed to determine the vgs of each prep. Primers used were 5′-TTC AAG GAC TTG GAG CCC AT-3′ and 5′-CCA CTT CTT GAG CAG GTC AG-3′ and the qPCR was performed at the following condition: 95 °C: 10 mins, followed by 40 cycles of 95 °C: 30 s and 60 °C: 60 s.

### Statistical analysis

Experiments were repeated at least thrice, and data represented as mean ± SD. Graph Pad Prism 7.0 (Graph Pad Software, Inc) was used to perform two-tailed t-test to test significance of differences observed between different groups. (*< 0.05, **< 0.01, ***< 0.005).

## Electronic supplementary material

Supplementary data
