# Supplementary material for: Synergistic and independent action of endogenous microRNAs 122a and 199a for post-transcriptional liver detargeting of gene vectors
Source: Sci Rep. 2018 Oct 19;8:15539. doi: 10.1038/s41598-018-33801-4 (PMC6195616; doi:10.1038/s41598-018-33801-4)
Supplement: Supplementary file 1 — Supplementary data [file 41598_2018_33801_MOESM1_ESM.docx]

**Synergistic and independent action of endogenous microRNAs 122a and 199a for post-transcriptional liver detargeting of gene vectors**

Bijay Dhungel^1, 2, 3^, Charmaine A. Ramlogan-Steel^2,4^, Jason C. Steel*^2, 4^

^1^Gallipoli Medical Research Institute, Greenslopes Private Hospital, 102 Newdegate Street, Brisbane, QLD 4120, QLD, AUS

^2^Faculty of Medicine, The University of Queensland, 288 Herston Road, Herston, Brisbane, QLD 4006, QLD, AUS

^3^University of Queensland Diamantina Institute, Translational Research Institute, 37 Kent Street, Woolloongabba QLD 4102, QLD, AUS

^4^OcuGene, Translational Research Institute, 37 Kent Street, Woolloongabba QLD 4102, QLD, AUS

Corresponding Author:

Jason C Steel

Faculty of Medicine, The University of Queensland, 288 Herston Road, Herston, Brisbane, QLD 4006, QLD, AUS

E-mail: [j.steel2@uq.edu.au](mailto:j.steel2@uq.edu.au)

Phone +61 7 33460611

Supplementary figure 1:

Supplementary figure 2:
